# Microbial melanins for radioprotection and bioremediation

**DOI:** 10.1111/1751-7915.12807

**Published:** 2017-08-14

**Authors:** Radames J. B. Cordero, Raghav Vij, Arturo Casadevall

**Affiliations:** ^1^ W. Harry Feinstone Department of Molecular Microbiology and Immunology Johns Hopkins Bloomberg School of Public Health 615 North Wolfe Street Baltimore MD 21205 USA

## Abstract

Microbial melanins provide a biocompatible and scalable approach for bioremediation and radioprotection technologies due to their physicochemical properties.

Sustainable development requires global stewardship which implements practices that ensure a healthy planet Earth and that repair existing environmental damage. Decades of anthropogenic pollution and the consequent depletion of Earth's ozone layer requires sustainable technologies and materials that can both neutralize toxic compounds from the environment and shield against increasing levels of harmful electromagnetic radiation (WHO 2003; Lelieveld *et al*., 2015). The creation and release of radioactive materials by the nuclear power and weapons industries present serious risks to ecosystems (Kyne and Bolin, 2016). Moreover, protection against cosmic ionizing radiation poses a huge challenge to interplanetary manned space travel and exploration (Durante and Cucinotta, 2011). Existing solutions to these problems are often expensive, unscalable and/or use materials that deplete natural resources. Here, we explore potential uses of microbial melanins as promising natural substances to be considered for bioremediation and radioprotection purposes owing to their biocompatibility, scalability and physicochemical properties.

Melanins are special multifunctional pigments normally found in animals, plants, fungi and bacteria (Solano, 2014). These biomolecules are structurally complex. They are derived by the oxidation of phenolic and/or indolic compounds that polymerize into ordered planar layers that aggregate into disordered macromolecular configurations (Watt *et al*., 2009). However, the primary and higher‐order structure of melanins translates into a combination of physicochemical properties uncommon in nature that includes: broad optical absorption and interaction with ionizing radiation, powerful antioxidant activity and binding affinity to a broad range of chemical compounds. Thus, in biology, melanization is associated with protection and adaptation to multiple chemical and mechanical stressors such as temperature, radiation, humidity and toxicity by different pollutants. The ecology of some melanotic microorganisms is noteworthy because they thrive in extreme environments (i.e. the Polar Regions, the damage nuclear reactor in Chernobyl and oil‐contaminated soil) and reviewed in ref. (Cordero and Casadevall, 2017). Melanin's broad absorption of electromagnetic energy combined with adsorption of radiation energy and chemicals respectively, make melanin‐producing microorganisms particularly useful for radioprotection and bioremediation processes (Revskaya *et al*., 2012; Gustavsson *et al*., 2016). Melanin biosynthesis in fungi has also been related to the assimilatory metabolism of priority pollutants (Prenafeta‐Boldú *et al*., 2006).

## Radioprotection

**Figure 1:**
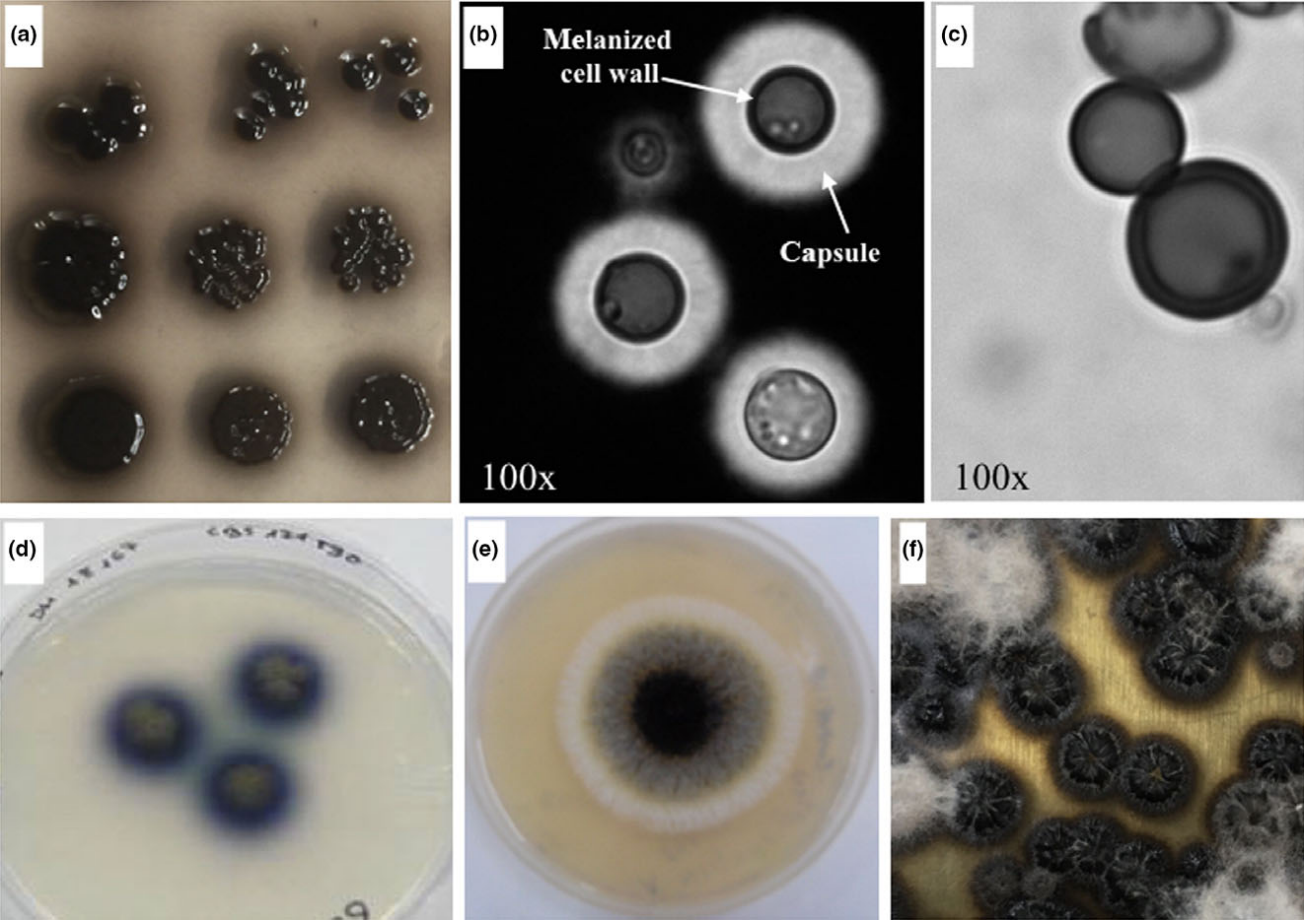
Examples of melanotic fungi. Melanized *C. neoformans* A. colonies (courtesy of Ricardo Perez Dulzaides), B. cells in India Ink suspension showing the black cell body and C. melanin ‘ghosts’ that remain after extensive enzyme and acid digestion. D. The black yeast, *Exophiala bergeri* grown in agar containing the chromogen ABTS (image courtesy of Dr. Francesc X. Prenafeta‐Boldú). E. *Aspergillus niger* grown on agar (image courtesy of Aashiyan Singh, Amity University 2014). F. Colonies of *Lomentospora prolificans* (courtesy of Nina Grossman).

Depending on the exposure and frequency, ionizing radiation can cause serious health problems and disrupt electronic devices (Johnston, 2000). Ionizing radiation constitutes high‐frequency electromagnetic waves (ultraviolet, gamma, X‐rays) or sub‐atomic particles (electrons, protons, neutrons, heavy metal ions) that contain enough energy to ionize or remove electrons from matter. Exposure to biological tissue can result in the generation of cytotoxic reactive oxygen species (ROS) that damage intracellular molecules (i.e. DNA, proteins). Studies in yeast have demonstrated that melanization can protect against various forms of ionizing radiation (Pacelli *et al*., 2017). Melanin mediates radioprotection by both (i) absorbing radiation energy and dissipating it in the form of heat while limiting the generation of ROS and/or by (ii) trapping and neutralizing the free radicals or ROS generated by the ionization of molecules (Dadachova *et al*., 2008; Khajo *et al*., 2011). These properties of melanin help explain how melanotic microorganisms can thrive or adapt in environments of extreme radiation and stimulate their evaluation as practical materials for radioprotection purposes (Cordero, 2017). Studies have already demonstrated that administering black fungi, isolated fungal melanin or synthetic melanins can successfully protect mammalian systems against ionizing radiation. A fungal melanin suspension isolated from *Cryptococcus neoformans* is capable of shielding X‐rays at a level similar to lead and two times more than charcoal (Dadachova *et al*., 2008). Although the radiation shielding performance of microbial melanins relative to that of existing shielding materials remains to be further evaluated, our current understanding suggests that microbial melanins may present several advantages over other technologies and materials, including protection against various forms of ionizing radiation, reduced toxicity in mammals and opportunities for large‐scale production (Fig. 1).

**Figure 2:**
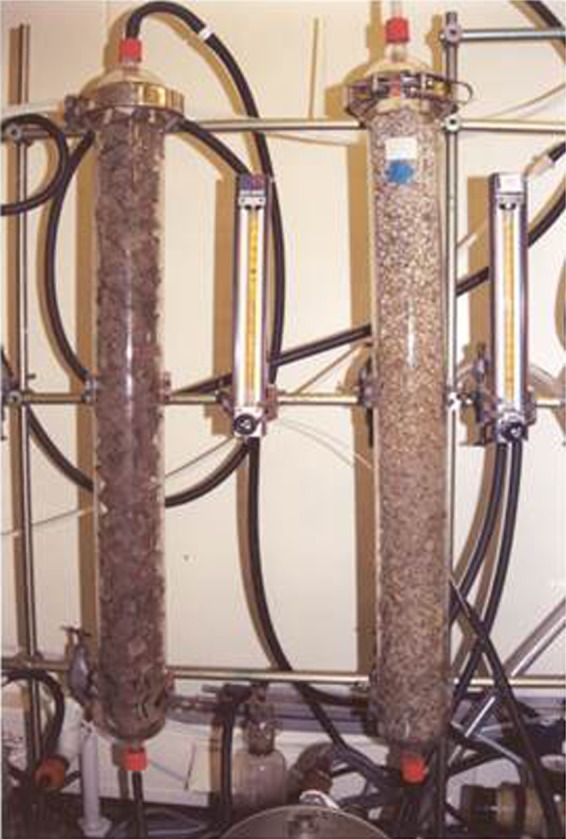
An example of a laboratory‐scale biofiltration system using *C. psammophila* as the biocatalyst for the elimination of toluene. The presence of the fungus can be observed by the darkening of polyurethane foam (*left*) and perlite (*right*) (Prenafeta‐Boldú *et al*., 2008). Image courtesy of Dr. Francesc X. Prenafeta‐Boldú.

## Bioremediation of volatile organic compounds

Emission of volatile organic compounds from industrial processes presents a major hazard and their removal from the environment is difficult and costly (Irvine and Sikdar, 1997). Sustainable biological processes such as gas biofiltration present several advantages over conventional methods (i.e. physicochemical adsorption, condensation, incineration) including the relatively lower costs of investment, performance and maintenance (van Groenestijn and Hesselink, 1993). Biofiltration consists of the ad/absorption and biodegradation of contaminants as the polluted air flows through a microbial biofilm (typically bacteria and/or fungi) immobilized in a solid medium (Fig. 2). Fungal‐based biofilters provide several advantages over bacterial ones including the ability to tolerate a wider range of environmental conditions that are difficult to control in a bioreactor with no free water (Estrada *et al*., 2013). Several black fungal species from the genera *Exophiala*,* Cladophialophora* and *Pseudallescheria* have been isolated from highly polluted areas (i.e. industrial spills) and can capture and degrade volatile aromatic hydrocarbons, including toluene, ethylbenzene and styrene (Prenafeta‐Boldú *et al*., 2006; Blasi *et al*., 2016). Furthermore, some species can use these compounds as their sole carbon source for growth (Weber *et al*., 1995; Prenafeta‐Boldú *et al*., 2001). The ability of these black fungal species to metabolize aromatic compounds is likely related to the biosynthesis and chemical structure of melanin, capable of interacting with a great variety of chemicals (Larsson, 1993). As melanins are resistant to acid hydrolysis and are highly hygroscopic, these melanotic species can tolerate acidic and dry conditions, making them attractive biocatalysts in air biofilters, as well as, in the *in‐situ* bioremediation of polluted soils in extreme environments.

## Bioremediation of metals

Metals, as elements, cannot be degraded or destroyed, thus they persist and accumulate in the environment and can pose a threat to human health and ecosystems. The methods commonly used for their removal including landfill treatment, acid leaching and electro‐reclamation are expensive and resource intensive (Mohammed *et al*., 2011), while the use of clay and minerals may be more cost‐effective materials capable of adsorbing heavy metals (Uddin, 2017). Numerous studies have shown the potential of using microorganisms for heavy metal bioremediation given their relative abundance in soils polluted with heavy metals (Kolesnikov *et al*., 2000). Among these, melanotic species are attractive given the remarkable propensity of melanins to react with different metals, a process often involving multiple coordination bonds between the carboxyl, amine and hydroxyl functional groups present in the pigment (Hong and Simon, 2007). Melanins are free radical biomolecules, therefore unpaired electrons can also contribute to the melanin‐metal interaction (Buszman *et al*., 2006). Black fungi exploit melanins for protection against metal toxicity and/or as a reservoir for physiologically required metals (Ban *et al*., 2012). Moreover, fungal melanins can efficiently adsorb heavy metals, including lead and zinc, in bioremediation of soil (Fogarty and Tobin, 1996). Relative to clay materials, dried biomass of melanized fungi resulted in 50–200 times more metal uptake in terms of surface area (Fomina and Gadd, 2003).

## Bioremediation radionuclides

Applications based on radionuclides pose risks to ecosystems and require innovative technologies for disposal of these substances and mitigate the effects of disasters. Existing methods to remove these pollutants from soils include adsorption using activated charcoal, membrane filtration methods and bioremediation technologies using plants, bacteria and/or fungi. Melanotic microorganisms are particularly attractive given their remarkable ability to grow in highly radioactive sites (Dighton *et al*., 2008) and the capacity of melanin pigments to readily adsorb radionuclides such as uranium and cobalt (McLean *et al*., 1998; Mahmoud, 2004). For instance, fungal melanin has a significantly greater capacity to adsorb uranium (~10‐fold) than activated carbon (Saini and Melo, 2013).

To conclude, the physicochemical properties of melanins combined with their abundance in the microbial world make these substances – and the microbes containing them – useful for achieving a sustainable future. There is now considerable evidence that these pigments can protect life from radiation damage and provide new tools for cleaning up the heavy metal and volatile hydrocarbons pollutants from the environment. One limitation is that melanins are associated with microbial virulence and a number of melanotic species are pathogenic to humans (Nosanchuk and Casadevall, 2003), restricting the pool of species and applications. However, melanin synthesis is widespread in the fungal kingdom and there are many species that produce melanin and are not pathogenic. Apart from limiting the use of non‐pathogenic species, possible mechanisms to overcome this limitation include the attenuation of virulence by genetic or chemical manipulations or the transformation of non‐pathogenic species with melanin production capacity. For example, *Escherichia coli* can be genetically engineered to melanize its surface and later remove pharmaceutical pollutants from wastewater with high efficiency (Gustavsson *et al*., 2016).

Melanins represent a largely unexploited set of compounds that likely prove versatile in many areas of environmental protection and remediation. Melanotic microorganisms may provide a valuable resource for various applications in support of the sustainable development goals 3, 6, 14 and 15 (UNDP 2015) by cleaning up the environment and protecting humans from pollutants and radiation damage.

## Conflict of Interest

None declared.
